# A Delay-Aware Switching Framework for Binary Actuator Systems Under Sensing Delay and Measurement Noise

**DOI:** 10.3390/s26144596

**Published:** 2026-07-20

**Authors:** Kiman Ji

**Affiliations:** Launch Vehicle Product Assurance Office, Space Launch Vehicle Research Directorate, Korea Aerospace Research Institute, Daejeon 34133, Republic of Korea; kmjee@kari.re.kr; Tel.: +82-10-4724-2545

**Keywords:** binary actuator systems, delay-aware switching, sensing delay, repeated triggering, ON/OFF control

## Abstract

**Highlights:**

**What are the main findings?**

- Repeated triggering in delayed binary actuator systems was formulated as a decision-timing problem caused by sensing delay and continuous threshold evaluation.
- The proposed delay-aware switching framework eliminated repeated triggering and reduced oscillation amplitude under delayed and noisy measurement conditions.

**What are the implications of the main findings?**

- Temporarily suspending switching-condition evaluation during the protected waiting phase can improve switching robustness without requiring a plant model.
- The proposed framework provides a practical approach for reliable ON/OFF actuator control in resource-constrained embedded systems such as Raspberry Pi-based automation platforms.

**Abstract:**

Binary actuator systems are widely used in industrial and agricultural automation because of their simple structure and low implementation cost. However, sensing delay and measurement noise can cause repeated triggering when threshold conditions are continuously evaluated using delayed feedback signals. This paper proposes a delay-aware switching framework for binary actuator systems operating under sensing uncertainty. The proposed framework structures the control cycle into sequential actuation, protected waiting, and idle phases. During the protected waiting phase, switching-condition evaluation is temporarily suspended to prevent redundant actuator commands caused by stale measurements. In addition, an adaptive supervisory mechanism updates the actuation duration and waiting interval using simple performance indicators obtained directly from sensor measurements, without requiring plant-model identification. The timing properties of the proposed switching sequence are analyzed, showing that the inter-event time is bounded below by a positive constant, thereby ensuring Zeno-free behavior. Simulation results demonstrate that the proposed framework eliminates repeated triggering and reduces oscillation amplitude under delayed and noisy measurements. Experimental validation using a Raspberry Pi-based thermal chamber further confirms bounded and regular switching behavior under artificially added measurement noise.

## 1. Introduction

Binary actuator systems are widely used in industrial and agricultural automation because of their simplicity, reliability, and low implementation cost. Representative applications include smart irrigation systems, greenhouse environmental control, HVAC systems, refrigeration equipment, and industrial fluid processes, where control actions are performed using ON/OFF actuators such as valves, pumps, heaters, and fans [1,2,3,4]. In such systems, control is typically achieved through threshold-based switching logic rather than continuously adjustable control inputs. Although binary control structures provide practical advantages in terms of implementation simplicity and robustness, they also introduce important operational challenges in modern sensor-based environments. In particular, sensing delay and measurement noise have become unavoidable due to the increasing use of low-cost sensors, wireless communication, filtering processes, and distributed monitoring systems. Furthermore, many practical sensing devices inherently contain measurement uncertainty and threshold fluctuations [5]. Under sensing delay and measurement noise, continuously evaluated threshold logic can repeatedly respond to stale or fluctuating measurements before the effect of the previous actuation becomes observable, especially when no explicit decision-hold or lockout mechanism is implemented [6]. This phenomenon, referred to in this paper as repeated triggering, leads to excessive actuator operation, increased mechanical wear, higher energy-related stress, and reduced system reliability.

Conventional continuous-control approaches, such as PID control and Smith predictor-based dead-time compensation, can be effective in many process-control applications. However, their direct use in low-cost practical ON/OFF actuator systems may require additional modulation, model information, or tuning effort, which limits their applicability when only simple binary switching commands are available [7,8]. In addition to tuning effort, model-based compensation requires an accurate representation of the plant dynamics and sensing delay. Obtaining such a model can be difficult in binary-actuated systems because ON/OFF excitation may provide limited input richness, while delayed and noisy measurements combine process dynamics, transport delay, sensor lag, and measurement uncertainty. Identification under binary or quantized observations generally requires specialized estimation methods and persistent-excitation conditions, and nonlinear identification is strongly affected by experiment design and operating-range coverage [9]. The difficulty is further increased by hysteresis, saturation, and piecewise nonlinearities; recent physics-informed sparse-identification studies show that model accuracy depends on the selected nonlinear basis, excitation type, numerical processing method, and noise robustness [10]. Therefore, obtaining and validating an accurate nonlinear delay model may impose substantial experimental and computational burden in low-cost binary-actuator applications.

In contrast, practical ON/OFF actuator systems operate under strict binary switching constraints, where the controller can only issue discrete actuation commands. Under delayed sensing conditions, this creates a delay-induced feedback mismatch between the actual plant state and the measurement, leading to severe actuator degradation and reduced operational lifetime [5]. In smart agriculture systems, such behavior may cause over-irrigation and inefficient resource utilization, whereas in HVAC and industrial thermal systems, it can degrade operational stability and energy efficiency. To mitigate rapid switching behavior, various switching strategies such as hysteresis control, dwell-time-based switching, and minimum-switching control have been investigated [11,12,13,14]. In addition, dwell-time constraints have been extensively investigated to guarantee stability in switched systems with delays [15,16,17], while event-triggered control approaches have been proposed to reduce unnecessary control updates and communication burden in networked systems [18,19,20,21,22,23,24]. Despite these advances, important limitations remain. Existing approaches mainly focus on limiting switching frequency or reducing communication activity, whereas the structural interaction between delayed feedback and continuous condition evaluation is not explicitly addressed. Many dwell-time-based implementations primarily constrain the admissible switching instants after a transition has occurred, while the timing of threshold-condition evaluation during the sensing-delay interval is often not explicitly separated from the measurement-update process. Consequently, the controller may repeatedly evaluate the same stale measurement during the sensing delay interval, resulting in redundant triggering events. Although event-triggered control has been extensively studied for delayed and networked systems, its primary objective is often to reduce communication or control-update activity under stability guarantees. In contrast, the present work focuses on a practical binary threshold-switching structure in which stale delayed measurements can repeatedly satisfy switching conditions unless the decision-evaluation interval itself is explicitly regulated [25,26]. Therefore, the fundamental issue in delayed binary actuator systems is not simply excessive switching frequency itself, but rather a decision-timing conflict caused by delayed sensing and continuously evaluated switching conditions. This issue cannot be fully resolved using conventional hysteresis or dwell-time approaches alone.

To address this limitation, this paper proposes a delay-aware switching control framework for binary actuator systems operating under sensing delay and measurement noise. Instead of relying on continuous threshold tracking, the proposed strategy governs decision timings through a discrete three-stage execution profile. This approach strategically decouples the measurement delay interval from the control logic by enforcing a temporary lockout on condition evaluations immediately after an actuation event. Unlike conventional hysteresis or dwell-time approaches that still continuously process delayed signals, the proposed methodology temporally isolates feedback latency from the underlying decision-making loop. As a result, redundant triggering caused by delayed and noisy measurements can be effectively suppressed while maintaining the structural simplicity of binary actuator control. In addition, the proposed framework incorporates an adaptive supervisory mechanism that updates the actuation duration and waiting interval using simple performance indicators obtained directly from sensor measurements. The adaptive mechanism does not require a plant model or parameter identification process and enables a practical trade-off between tracking performance, overshoot characteristics, and switching robustness.

The effectiveness of the proposed framework is validated through both simulation and experimental studies. Simulations under delayed and noisy measurement environments demonstrate that the proposed method suppresses repeated triggering and significantly reduces oscillation amplitude compared with conventional hysteresis control. Experimental validation using a Raspberry Pi-based thermal chamber further confirms that the proposed framework maintains stable and regular switching behavior even under artificially injected measurement noise.

The main contributions of this paper are summarized as follows:

- Repeated triggering caused by delayed sensing and continuous threshold evaluation is formulated as a structural problem in binary actuator systems, distinct from the switching-frequency problem addressed by conventional hysteresis or dwell-time methods;
- A multi-phase chronological switching framework is established to systematically regulate the exact timing of threshold evaluations;
- A practical supervisory tuning mechanism is incorporated to adjust the actuation duration and waiting interval using measured output indicators, without requiring a plant model;
- The timing properties of the switching sequence are analyzed, including a positive minimum inter-event time and Zeno-free behavior;
- Simulation and experimental results, including validation using a Raspberry Pi-based thermal chamber, show that the proposed framework suppresses repeated triggering and maintains regular switching behavior under delayed and noisy measurements.

## 2. System Modeling and Formulation

### 2.1. Mathematical Modeling of Binary Actuator Systems

Binary actuator systems operate using discrete ON/OFF control inputs rather than continuously variable commands. Let *u*(*t*) ∈ {0, 1} denote the control input applied to the actuator, where *u*(*t*) = 1 and *u*(*t*) = 0 represent the active (ON) and inactive (OFF) states, respectively. The physical output of the plant is denoted by *y*(*t*). In practical industrial and agricultural infrastructures, the measured feedback signal is affected by sensing latency and measurement noise. The delayed state baseline, *y_s_*(*t*), is modeled as follows:

(1) $$y_{s}\left(t\right)=y\left(t-\tau \right),$$

where *τ* represents the total lumped sensing delay, encompassing sensor transduction dynamics, communication latency, and signal filtering intervals. Furthermore, the actual signal available to the controller, *y_m_*(*t*), includes high-frequency measurement noise *n*(*t*):

(2) $$y_{m}\left(t\right)=y_{s}\left(t\right)+n\left(t\right).$$

The switching decision is governed by a threshold-based policy σ(⋅). For the heating-type binary actuator considered in this study, the policy is defined as

(3) $$\begin{array}{cccc} & \sigma (y_{m}\left(t\right))=\left\{\begin{array}{cc} 1,\;\; & y_{m}\left(t\right)<\theta \\ 0,\;\; & y_{m}\left(t\right)\geq \theta \end{array}\right., & & \end{array}$$

where *θ* is the target threshold. Accordingly, the actuator is activated when the measured output is below the threshold and deactivated when the measured output reaches or exceeds it. For a cooling-type actuator, the inequality directions can be reversed. It should be emphasized that the proposed framework does not alter this threshold policy itself; instead, it regulates when the policy is evaluated. Specifically, *σ*(⋅) is evaluated only during the idle phase and is disabled during the actuation and protected waiting phases.

### 2.2. Mechanism of Repeated Triggering Under Sensing Uncertainty

**Figure 1:**
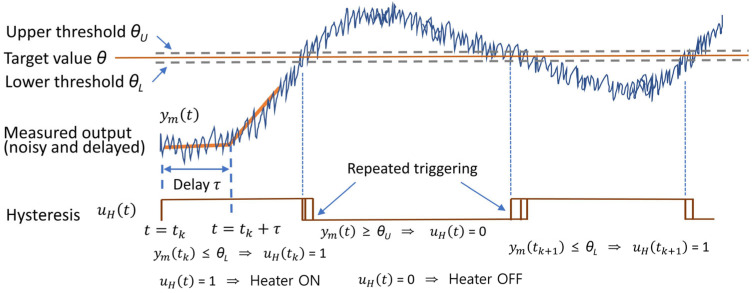
Relationship among the delayed and noisy measurement *y_m_*(*t*), the hysteresis control input $u_{H}(t)$, and repeated triggering under sensing delay.

In delayed binary control structures, the primary operational degradation arises from a mismatch between continuous threshold evaluation and the physical propagation delay *τ*. This mismatch becomes more pronounced in the presence of measurement noise. Figure 1 conceptually illustrates the relationship among the delayed and noisy measured signal *y_m_*(*t*), the hysteresis control input *u_H_*(*t*), and the resulting repeated switching events. When a state transition occurs at *t* = *t_k_*, the physical consequence of the actuator command does not become observable in *y_m_*(*t*) until approximately *t* = *t_k_* + *τ*.

During this unobservable interval [*t_k_*, *t_k_* + τ], the controller continuously evaluates the threshold conditions based on the stale measurement *y_m_*(*t*), which fails to reflect the current actuator state $u_{H}(t)$. In the presence of measurement noise *n*(*t*), this stale signal frequently crosses the control thresholds prematurely. This logical inconsistency forces the controller to issue multiple redundant and high-frequency switching commands before the plant response can stabilize the loop.

### 2.3. Operational Limitations of Conventional Control Strategies

To mitigate noise-induced instabilities, conventional frameworks typically rely on fixed hysteresis bands or heuristic dwell-time constraints. However, as summarized in Table 1, these approaches exhibit significant limitations when sensing delays and measurement noise are simultaneously present.

Standard hysteresis controllers effectively filter high-frequency noise *n*(*t*) by expanding the threshold margin. However, hysteresis bands do not compensate for sensing delays; if τ is large, the stale signal resides within the threshold zone long enough to execute multiple invalid transitions, rendering hysteresis bands ineffective against delay-driven oscillations. Alternatively, dwell-time control enforces a fixed blanking interval to prevent rapid switching. Although this approach can suppress repeated triggering, it introduces substantial empirical tuning overhead and may significantly degrade transient tracking performance. If the dwell time is set too short, it may fail to cover the worst-case latency τ; if it is set too long, it can cause excessive overshoot and reduce system responsiveness under time-varying thermal or hydraulic loads [27,28]. Moreover, after the dwell-time interval expires, switching decisions are again made based on the available measured signal, which may still be delayed relative to the actual plant state.

## 3. Proposed Delay-Aware Switching Framework

### 3.1. Framework Overview

**Figure 2:**
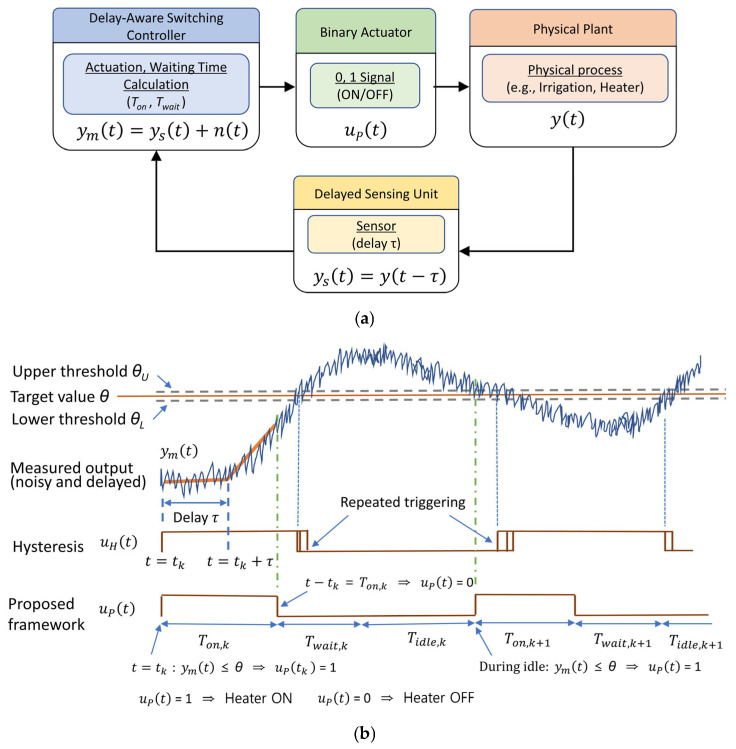
Proposed delay-aware switching framework: (**a**) schematic configuration of the binary-actuator system under delayed and noisy measurement; (**b**) relationship among the measured output *y_m_*(*t*), switching commands, and the actuation, protected waiting, and idle phases.

To address the delay-induced repeated-triggering mechanism described in Section 2, the proposed delay-aware switching framework is introduced in this section. Figure 2a shows the feedback configuration of the system under study, consisting of the delay-aware controller, binary actuator, physical plant, and delayed sensing unit. Figure 2b illustrates the decision-timing philosophy of the proposed framework and its relationship to the measured output *y_m_*(*t*). Unlike conventional hysteresis control, in which threshold conditions are continuously evaluated, the proposed framework divides each control cycle into actuation, protected waiting, and idle phases. Switching-condition evaluation is disabled during the actuation and protected waiting phases and is re-enabled only during the idle phase. A new actuation cycle begins when the threshold condition is satisfied during the idle phase. This structure differs from conventional fixed dwell-time control by separately defining a finite actuation interval *T_on,k_*, a delay-protection interval *T_wait,k_*, and a state-dependent idle interval *T_idle,k_*, rather than imposing a single fixed residence-time constraint. In addition, *T_on,k_* and *T_wait,k_* are adaptively updated according to the measured cycle performance.

Let *t_k_* denote the start time of the *k*-th actuation cycle, i.e., the instant at which the actuator changes from OFF to ON. The control input is defined as:

(4) $$\begin{array}{cccc} & u_{P}\left(t\right)=\left\{\begin{array}{cc} 1,\;\; & {\;\;\;\;\;t}_{k}\leq t<t_{k}+T_{on,k}\\ 0,\;\; & t_{k}+T_{on,k}\;\leq \;t\;<\;t_{k}+T_{on,k}+T_{wait,k}+T_{idle,k} \end{array}\right., & & \end{array}$$

where *T_idle,k_* is not a prescribed design parameter. It is a state-dependent monitoring interval that begins after the actuation and protected waiting phases and ends when the switching condition is satisfied again. Physically, *T_idle,k_* represents the time required for the measured output to return to the region in which a new actuation is necessary. If the switching condition is already satisfied at the end of the protected waiting phase, then *T_idle,k_* = 0; otherwise, the controller remains in the idle phase and monitors *y_m_*(*t*) until the condition is met. The next switching event satisfies:

(5) $$\begin{array}{cccc} & t_{k+1}=t_{k}+T_{on,k}+T_{wait,k}+T_{idle,k}. & & \end{array}$$

This guarantees that the inter-event time is always strictly positive, thereby preventing rapid repeated switching. Because the controller operates based on delayed measurements, as described in (1) and (2), the same switching condition may be repeatedly satisfied before the system response becomes observable. To prevent this phenomenon, the following design condition is introduced.

(6) $$\begin{array}{cccc} & T_{wait}\geq \tau . & & \end{array}$$

This ensures that switching decisions are evaluated only after the effect of the previous actuation has been reflected in the measured signal. The proposed framework can therefore be interpreted not as a modification of the switching condition itself, but rather as a temporal regulation mechanism that controls the timing of switching decisions.

### 3.2. Adaptive Supervisory Tuning of Dwell-Time Parameters

To reduce the need for repeated empirical tuning, a simple adaptive supervisory mechanism is introduced. After each cycle, the controller updates *T_on_* and the waiting time *T_wait_* based on the observed system response to improve tracking performance while limiting overshoot. The proposed update mechanism utilizes the following two performance indicators:

- Average-output tracking error;
- Overshoot magnitude.

These indicators provide intuitive information regarding the quality of the system response and can be computed without requiring a plant model. To drive the cycle-average output toward the target value, the proposed framework updates the actuation duration *T_on_* based on the cycle-average output rather than the instantaneous endpoint value.

For the *k*-th cycle, the cycle-average output is defined as

(7) $$y_{avg,k}=\frac{1}{T_{k}}\int_{t_{k}}^{t_{k}+T_{k}}y_{m}(t)dt,$$

where *T_k_* is the cycle duration. Since each cycle consists of an actuation, waiting and idle time, the cycle length is given by

(8) $$T_{k}=T_{on,\;k}+T_{wait,\;k}+T_{idle,\;k}$$

Let *θ* denote the reference value, then the average-output tracking error is defined as

(9) $$e_{k}=\theta -y_{avg,\;k}$$

The sign of the tracking error indicates the control outcome. If *e_k_* > 0, the output remains below the reference value and the previous actuation was insufficient. If *e_k_* < 0, the output has exceeded the reference value, indicating overshoot. To quantify transient violation beyond the reference value, the overshoot magnitude is defined as

(10) $${OS}_{k}=\max\left(0,\;y_{max,k}-\theta \right),$$

where *y*_max,*k*_ denotes the maximum output observed during the *k*-th cycle. These performance metrics provide simple indicators of regulation quality and can be evaluated directly from sensor measurements without requiring model-based estimation. The proposed update structure follows established adaptive tuning principles by incorporating bounded parameter updates, low-pass smoothing, and projection-based feasibility constraints.

#### 3.2.1. Step I: Parameter Update

Using the average-output tracking error, the intermediate update law for the actuation duration is defined as follows:

(11) $${\tilde{T}}_{on,k+1}=T_{on,k}+\alpha e_{k},$$

where α > 0 is the adaptation gain for the actuation duration. The waiting interval *T_wait_* is adjusted based on the overshoot magnitude. The waiting-time update is constructed as

(12) $${\tilde{T}}_{wait,k+1}=T_{wait,k}+\Delta T_{wait,k},$$

with

(13) $$\Delta T_{\mathrm{wait,k}}=\left\{\begin{array}{cc} \beta_{1}\left({OS}_{k}-{OS}_{ref}\right), & {OS}_{k}>{OS}_{ref},\\ -\beta_{2}e_{k}, & {OS}_{k}\leq {OS}_{\mathrm{ref}},\;|e_{k}>0,\\ 0, & \mathrm{otherwise}, \end{array}\right.$$

where *OS*_ref_ denotes the admissible overshoot level, and *β*_1_ > 0 and *β*_2_ > 0 are adaptation gains for increasing and decreasing the waiting time, respectively. To prevent excessive parameter variation and to preserve implementability, the updated dwell-time parameters are constrained within predefined bounds. With these update laws, the roles of the two adaptive parameters become clearly separated: *T_on_* regulates the center of the oscillatory response relative to the target, while *T_wait_* regulates the oscillation amplitude and prevents excessively rapid retriggering. The adaptive supervisor improves control performance while preserving the timing feasibility and delay-protection structure of the proposed framework.

#### 3.2.2. Step II: Low-Pass Smoothing

To prevent oscillatory parameter variations caused by measurement noise or cycle-to-cycle fluctuations, a first-order smoothing filter is applied.

(14) $$\begin{array}{cccc} & {\overset{-}{T}}_{on,k+1}=\left(1-\lambda \right)T_{on,k}+\lambda {\tilde{T}}_{on,k+1}, & & \end{array}$$

(15) $$\begin{array}{cccc} & {\overset{-}{T}}_{wait,k+1}=\left(1-\lambda \right)T_{wait,k}+\lambda {\tilde{T}}_{wait,k+1}, & & \end{array}$$

where 0 < λ ≤ 1 denotes the smoothing factor.

#### 3.2.3. Step III: Projection with Feasibility and Delay-Protection Constraints

The updated dwell-time parameters are then projected onto predefined feasible intervals to preserve timing feasibility and delay protection. Let $T_{on,min}$ and $T_{on,max}$ denote the minimum and maximum allowable actuation durations, respectively, and let $T_{wait,min}$ and $T_{wait,max}$ denote the minimum and maximum allowable waiting times. These bounds define the admissible range of the dwell-time parameters as follows:

(16) $$T_{on,min}\leq T_{on}\leq T_{on,max},\;\;\;T_{wait,min}\leq T_{wait}\leq T_{wait,max}$$

The dwell-time parameters are therefore projected onto these admissible intervals:

(17) $$\begin{array}{c} T_{on,k+1}=\Pi_{\left[T_{on,min},T_{on,max}\right]}\left({\overset{-}{T}}_{on,k+1}\right), \end{array}$$

(18) $$T_{\mathrm{wait,k}+1}=\Pi_{\left[\max(T_{\mathrm{wait,min}},\tau ),\;T_{\mathrm{wait,max}}\right]}\;\left(\max\left({\overset{-}{T}}_{wait,k+1},\tau \right)\right),$$

(19) $$\begin{array}{cccc} & T_{wait,max}\geq max(T_{wait,min},\tau ), & & \end{array}$$

where the projection operator

(20) $$\Pi_{\left[a,b\right]}\left(x\right)=\min\left(\max\left(x,a\right),b\right)$$

maps a candidate parameter value onto the admissible interval [*a*, *b*]. The projection operators ensure that the updated parameters remain within feasible bounds while simultaneously satisfying the delay protection constraint of Equation (6). Consequently, the proposed adaptive tuning structure provides robust delay protection without relying on plant identification or intensive optimization processes. This numerical simplicity enables reliable real-time execution with minimal computational and hardware burden, making it highly suited for industrial automation systems operating with strict timing limits.

#### 3.2.4. General Parameter-Selection Guidelines

The numerical values of the control parameters depend on the actuator capacity, process dynamics, sensing characteristics, and required control performance. Nevertheless, the parameters can be selected using a common procedure based on quantities that are directly measurable from a preliminary response test.

First, the effective sensing delay τ is determined as the elapsed time between an actuator-state transition and the first sustained observable response in the measured output. The minimum waiting time should then be selected so that the effect of the previous actuation is reflected in the measurement before the switching condition is evaluated again:

(21) $$T_{wait,min}\geq \tau +{∆}_{\tau }$$

where Δ_τ_ is a safety margin accounting for sampling time, delay uncertainty, measurement noise, and variations in the response-detection threshold. If the measured delay is sufficiently repeatable, Δ_τ_ may be small. For systems with greater delay uncertainty, a larger margin should be used.

Second, the initial actuation duration *T_on,_*_0_ can be determined from a short preliminary actuation test. Let *K_act_* denote the average output-change rate produced by the actuator over the relevant operating range. A practical initial estimate is

(22) $$T_{on,0}\approx \frac{∆y_{des}}{K_{act}}$$

where Δy_des_ denotes the desired output change during one actuation cycle. A smaller value of Δy_des_ results in finer regulation and lower overshoot, whereas a larger value provides faster convergence but may increase the oscillation amplitude.

The lower bound *T_on,min_* should be greater than the minimum actuator pulse that produces a measurable output response. The upper bound *T_on,max_* should be selected as the longest actuation duration that does not produce unacceptable overshoot or violate actuator and process constraints. Similarly, *T_wait,max_* should be selected as the longest inactive interval that still preserves the required responsiveness. These bounds should be determined from hardware limitations, allowable output variation, and preliminary step-response measurements.

The adaptation gain *α* determines the correction rate of the actuation duration. A larger *α* accelerates the movement of the cycle-average output toward the reference value, but an excessively large value may cause cycle-to-cycle variation in *T_on_*. Therefore, *α* should initially be selected conservatively and gradually increased until the desired convergence speed is obtained without oscillatory parameter updates.

The gains *β*_1_ and *β*_2_ determine the adjustment rate of the waiting interval. The gain *β*_1_ governs the increase in *T_wait_* when the observed overshoot exceeds the admissible level, whereas *β*_2_ governs the decrease in *T_wait_* when the actuation is insufficient and a faster response is required. Larger values provide faster correction, but excessively large values may cause abrupt variation in the waiting interval. These gains should therefore be tuned so that the waiting time changes gradually while maintaining the delay-protection condition.

The smoothing factor *λ* determines the balance between adaptation speed and noise sensitivity. A smaller *λ* provides stronger smoothing and reduces cycle-to-cycle parameter fluctuations, whereas a larger *λ* allows faster adaptation but increases sensitivity to measurement noise and transient disturbances. In processes with noisy measurements or slowly varying dynamics, a smaller value is generally preferable. In relatively repeatable and low-noise processes, a larger value may be used.

Finally, the admissible overshoot level *OS_ref_* should be selected from the allowable process-output deviation or application-specific safety requirement. The projection bounds should then be verified to ensure that all updated values satisfy both hardware feasibility and the delay-protection constraint.

The delay-normalized parameter values should be regarded as practical initialization rules rather than universal theoretical relationships. In particular, the waiting interval is directly related to the sensing delay, whereas the actuation duration, adaptation gains, smoothing factor, and parameter bounds are primarily determined by actuator strength, process-response characteristics, allowable overshoot, desired convergence speed, and measurement-noise sensitivity.

#### 3.2.5. Boundedness and Local Conditional Convergence of the Adaptive Updates

The following analysis establishes boundedness and conditional local convergence of the proposed cycle-to-cycle parameter updates. The adaptive mechanism may be interpreted as a low-complexity supervisory law related to P-type iterative learning control; however, it updates only two scalar timing parameters and does not require trajectory learning, periodic perturbation, or explicit gradient estimation.

The projection operators guarantee that, for every cycle k,

(23) $$T_{on,k}\in \left[T_{on,min},T_{on,max}\right],\;$$

(24) $$T_{wait,k}\in \left[{max(T}_{wait,min},{\tau ),\;T}_{wait,max}\right],$$

thereby preventing parameter drift and preserving the feasibility and delay-protection constraints. Projection alone, however, guarantees boundedness rather than convergence to a unique equilibrium. To examine the local behavior of the actuation-duration update, the waiting-time parameter and operating condition are regarded as locally fixed. Under this condition, the noise-free cycle-average output can be represented locally as

(25) $${\overset{-}{y}}_{k}=F(T_{on,k}),$$

where F(⋅) is assumed to be continuous and monotonically increasing over the admissible interval. When the projection is inactive, the update and smoothing equations can be combined as

(26) $$T_{on,k+1}=T_{on,k}+\lambda \alpha \left[\theta -F(T_{on,k})\right].$$

Let $T_{on}^{*}$ be a feasible equilibrium satisfying

(27) $$F(T_{on}^{*})=\theta ,$$

and define the parameter error as

(28) $${\tilde{T}}_{k}=T_{on,k}-T_{on}^{*}.$$

Assume that *F* is differentiable and that its local sensitivity satisfies

(29) $$0<m\leq F^{\prime }(T_{on})\leq M,$$

where m  and M  denote the minimum and maximum local sensitivities of the cycle-average output to the actuation duration. By the mean-value theorem,

(30) $${\tilde{T}}_{k+1}=\left[1-\lambda \alpha F^{\prime }(\xi_{k})\right]{\tilde{T}}_{k},$$

where $\xi_{k}$ lies between $T_{on,k}$ and $T_{on}^{*}$. Therefore, the error decreases if

(31) $$|1-\lambda \alpha F^{\prime }(\xi_{k})|<1.$$

This condition is equivalent to

(32) $$0<\lambda \alpha F^{\prime }(\xi_{k})<2.$$

Since $F^{\prime }(\xi_{k})\leq M$, a sufficient condition over the local admissible interval is

(33) $$0<\lambda \alpha <\frac{2}{M}.$$

Under this condition, $T_{on,k}$ converges locally toward $T_{on}^{*}$, provided that the equilibrium lies within the admissible interval and the local monotonicity assumption remains valid. If the required equilibrium lies outside the feasible interval, the projection operator drives the parameter toward the nearest admissible boundary. To obtain an experimental estimate of the local sensitivity, the previously recorded cycle data were analyzed. For two adjacent operating cycles with different actuation durations, the finite-difference sensitivity was calculated as

(34) $$\hat{F^{\prime }}=\frac{\bar{y}\left(T_{on,2}\right)-\bar{y}\left(T_{on,1}\right)}{T_{on,2}-T_{on,1}},$$

where $\bar{y}\left(T_{on,i}\right)$ denotes the time-weighted cycle-average temperature corresponding to *T_on,i_*. To avoid distortion caused by the initial warm-up transient and premature termination of the final cycle, the initial transient pair and the incomplete final cycle were excluded from the sensitivity-bound evaluation. The maximum positive sensitivity obtained from the remaining complete cycles was $\max\hat{F^{\prime }}\approx 0.020135\;℃/s.$ Considering measurement variation, residual thermal effects, and cycle-to-cycle disturbances, the upper sensitivity bound was conservatively selected as M=0.025 ℃/s. This value is larger than the maximum experimentally estimated local sensitivity and therefore provides a safety margin for evaluating the adaptation gains. The corresponding sufficient convergence limit is $2/M=80\;s{/}^{\circ }C.$ For the fan-ON condition, $\lambda \alpha =0.6\times 25=15\;s{/}^{\circ }C,$ while for the fan-OFF condition, $\lambda \alpha =0.8\times 40=32\;s{/}^{\circ }C.$ Both values satisfy $0<\lambda \alpha <80\;s{/}^{\circ }C$ and are therefore consistent with the derived sufficient local-convergence condition.

The waiting-time update can be interpreted similarly. Let

(35) $$OS_{k}=G(T_{wait,k}),$$

where G(⋅) is assumed to be continuous and non-increasing over the admissible interval. This assumption reflects the tendency of a longer waiting interval to reduce premature retriggering and excessive overshoot. The update therefore moves $T_{wait,k}$ toward the admissible set

(36) $$S_{OS}=\left\{T_{wait}:G(T_{wait})\leq OS_{ref}\right\}.$$

Because the waiting-time law is piecewise and affected by both overshoot and response sufficiency, convergence is interpreted as convergence toward an admissible set rather than toward a unique value.

In the presence of measurement noise, sensing-delay variations, residual thermal effects, and cycle-to-cycle disturbances, exact convergence to a single parameter value cannot generally be guaranteed. Instead, smoothing and projection yield bounded parameter trajectories and practical convergence to a neighborhood of the feasible equilibrium set. Accordingly, the present analysis establishes boundedness and conditional local convergence rather than unconditional global convergence.

### 3.3. Structural Timing Properties

According to the proposed switching cycle, the actuator remains active during the actuation phase of duration *T_on_*, followed by the waiting phase of duration *T_wait_*. Since switching conditions are not evaluated during these intervals, the earliest possible occurrence of the next actuation-cycle initiation event is given by

(37) $$t_{k+1}-t_{k}\geq T_{on,min}+max(T_{wait,min},\tau ).$$

The proposed timing structure guarantees a positive minimum inter-cycle interval, thereby preventing Zeno-like rapid switching behavior. The minimum inter-cycle interval also provides an upper bound on the switching frequency. Let *N*_cyc_(*T*) denote activation-cycle count within the interval [0, *T*]. Then,

(38) $$\begin{array}{cccc} & N_{cyc}\left(T\right)\leq \left[\frac{T}{T_{on,min}+\max\left(T_{wait,min},\tau \right)}\right]+1. & & \end{array}$$

Accordingly, the maximum activation-cycle frequency satisfies

(39) $$\begin{array}{cccc} & f_{cyc,max}\leq \frac{1}{T_{on,min}+\max\left(T_{wait,min},\tau \right)}. & & \end{array}$$

The dwell-time parameters determine the activation-cycle frequency and minimum inter-cycle interval.

## 4. Simulation Results

### 4.1. Simulation Setup and Evaluation Metrics

To evaluate the effectiveness of the proposed delay-aware switching strategy, simulations were performed using a simplified thermal-process model incorporating adjustable sensing delay and measurement noise. The mock thermal system emulates a temperature regulation process driven by a binary heater input. Let *y*(*t*) denote the true plant temperature, *y_a_* the ambient temperature, and *u*(*t*) the heater command. The plant dynamics were implemented in discrete time as

(40) $$y\left(t+∆t\right)=y\left(t\right)+\left(K_{h}u\left(t\right)-k_{c}\left(y\left(t\right)-y_{a}\right)\right)∆t,$$

where *K_h_* is the heating gain and *k_c_* is the passive cooling coefficient. Heating increases the plant temperature when the binary actuator is ON, whereas passive cooling decreases the temperature toward the ambient condition when the actuator is OFF. In the simulation code, the heating gain and cooling coefficient were set to (*K_h_* = 0.5 °C/s) and (*k_c_* = 0.01 s^−1^), respectively. No process disturbance term was applied in the simulation. Sensing delay and Gaussian measurement noise were added to the simulated sensor output to emulate realistic measurement uncertainty, and the controller used only the delayed and noisy measurement for switching decisions. All simulations were conducted under the following conditions:

- Target temperature: 35 °C;
- Ambient temperature: 20 °C;
- Simulation duration: 600 s;
- Sampling time: 0.2 s.

The parameters for each control method were configured as summarized in Table 2. Delay-dependent parameters were expressed as functions of the sensor delay τ.

In Table 2, *α*, *β*_1_, *β*_2_, and λ are tuning parameters selected according to the heater capacity and heat-transfer characteristics of the thermal system. Specifically, α determines the convergence speed of T_on_, β_1_ increases T_wait_ when the overshoot is excessive, β_2_ decreases T_wait_ to improve responsiveness, and λ suppresses abrupt parameter variations caused by measurement noise.

### 4.2. Comparison of Output Response and Switching Behavior

**Figure 3:**
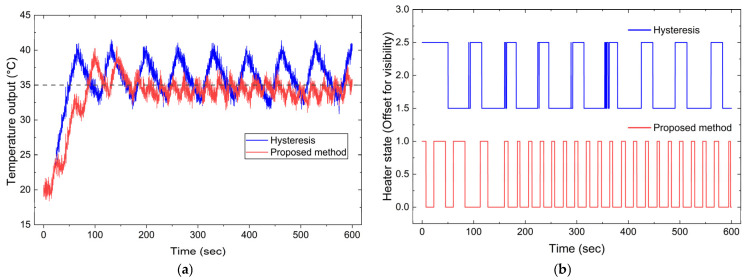
Output responses and switching behaviors of the hysteresis control and the proposed framework: (**a**) Output responses; (**b**) Switching behaviors under delayed and noisy measurements.

Simulations were conducted to analyze the response characteristics and repeated triggering characteristics of the conventional hysteresis control method and the proposed method under delayed and noisy measurement environments. Figure 3a shows the output responses of the conventional hysteresis controller with a hysteresis band of ±1 °C and the proposed control method when control decisions are made based on measurements containing a delay of 15 s and noise with a standard deviation of 0.7 °C. Figure 3b illustrates the difference in switching characteristics between the hysteresis controller and the proposed framework.

As shown in Figure 3b, the hysteresis method and the proposed method exhibit different switching characteristics. The simulation results are summarized in Table 3. The repeated triggering ratio represents the proportion of redundant actuator switching events relative to the total number of switching transitions. Although the proposed framework exhibits a higher number of switching transitions than the hysteresis controller, no repeated triggering occurred, and the oscillation amplitude was smaller, indicating improved convergence characteristics. Since the heater operates as a binary actuator with constant power consumption during the ON state, the accumulated-ON duration can be interpreted as a normalized energy consumption metric. The proposed method reduced the total actuator ON-time from 230.3 s to 164.4 s, corresponding to approximately 28.6% lower normalized ON-time for the specific simulated operating condition. This result should not be interpreted as a general guarantee of reduced energy consumption.

**Figure 4:**
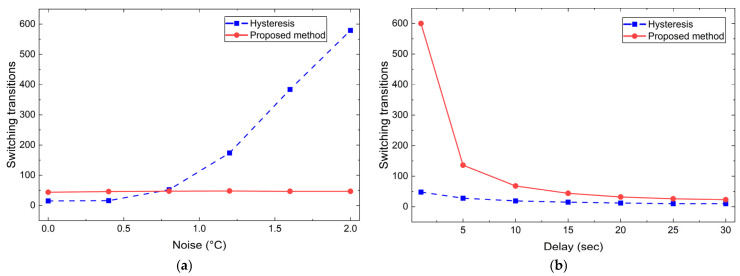
Switching activity under sensing uncertainty: (**a**) Switching transitions versus measurement noise; (**b**) Switching transitions versus sensing delay.

The hysteresis controller exhibits frequent and irregular switching due to repeated threshold crossings caused by measurement noise. This phenomenon is not caused by plant instability, but rather by the delay-induced feedback mismatch between delayed sensing and continuous threshold evaluation. In practical systems, such behavior leads to unnecessary actuator wear, increased energy consumption, and degradation of system reliability. In contrast, the proposed framework maintains well-defined and regular switching patterns. Since switching decisions are evaluated only at discrete cycle boundaries, instantaneous noise fluctuations do not directly trigger switching events. The proposed framework does not primarily aim to minimize switching count itself, but rather to eliminate redundant switching events caused by delayed feedback. Figure 4 quantitatively compares the number of switching transitions, where both ON-to-OFF and OFF-to-ON changes are counted as switching transitions.

As shown in Figure 4a, the switching count of the hysteresis controller increases rapidly as the measurement noise increases. In contrast, the proposed method maintains an almost constant switching count, demonstrating strong robustness against sensing uncertainty. Figure 4b shows that, as the sensing delay increases, the switching count of the proposed framework decreases and approaches the range of the hysteresis controller. In the hysteresis controller, the reduction in switching frequency leads to increased output oscillation amplitude, which may result in degraded control performance due to overheating and excessive temperature fluctuations.

### 4.3. Delay-Dependent Oscillation Amplitude

**Figure 5:**
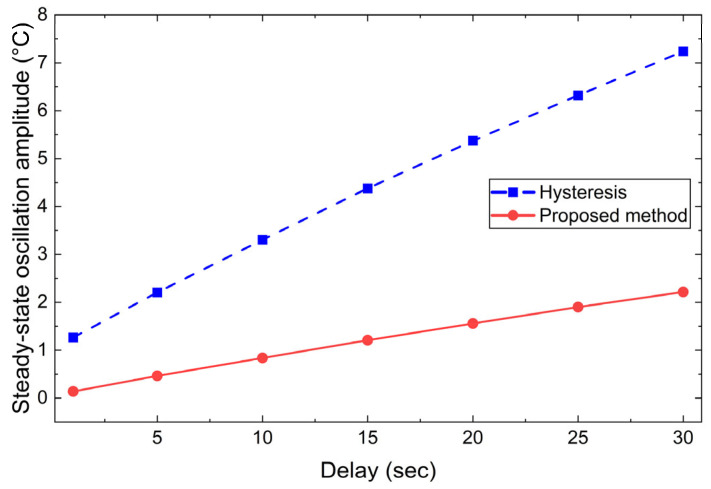
Oscillation amplitude versus sensing delay.

The relationship between process measurement delay and the output oscillation amplitude of each controller was analyzed. Figure 5 shows the oscillation amplitude of the output in the steady state as the sensing delay increases. The hysteresis controller exhibits a clear monotonic increase in oscillation amplitude as the sensor delay increases. As the delay becomes larger, switching decisions are increasingly based on outdated measurements, resulting in delayed and excessive actuation that significantly amplifies temperature oscillations. In contrast, although the proposed control method also shows an increasing trend, its growth rate is significantly lower.

This indicates that the proposed delay-aware switching framework effectively mitigates delay-induced control errors. Such large oscillations are undesirable because they not only degrade control quality, but also reduce energy efficiency and may induce instability in sensitive industrial processes. In practical systems, maintaining bounded switching activity is critically important for extending actuator lifetime and reducing maintenance costs. The proposed delay-aware control method achieves this by explicitly regulating switching timing, thereby ensuring stable and predictable actuator behavior.

## 5. Experimental Results

### 5.1. Test Setup

**Figure 6:**
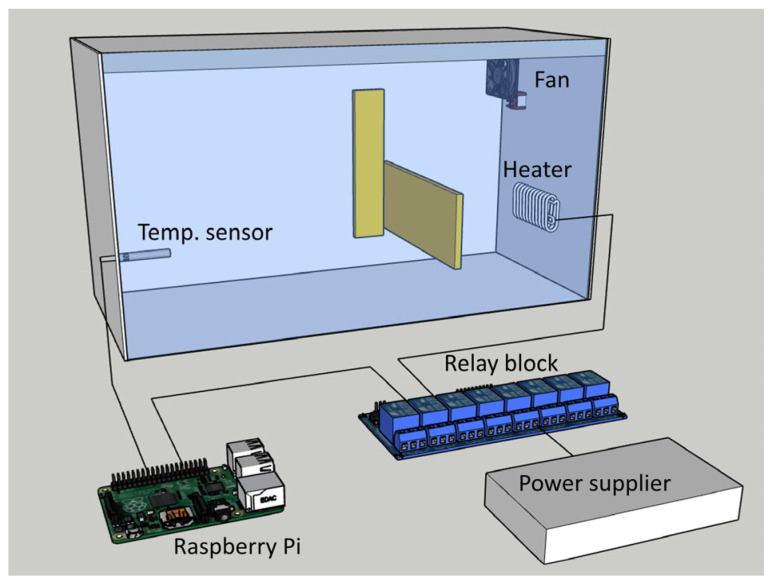
Temperature chamber and connection diagram of the experimental components.

**Figure 7:**
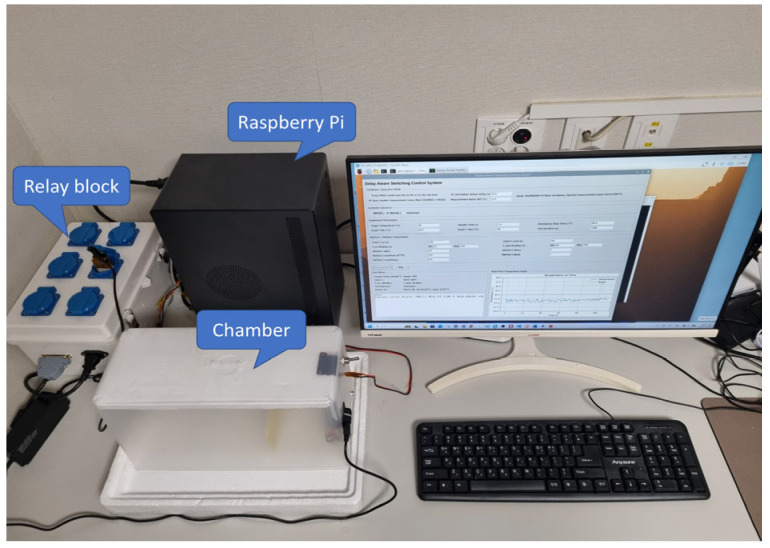
Experimental setup.

To validate the proposed framework, experiments were conducted using a small box-shaped chamber designed to maintain the temperature at 30 °C. Figure 6 illustrates the interior of the temperature chamber and the connection diagram of the experimental components. To limit direct heat transfer from the heater, a shielding plate was installed between the heater and the temperature sensor. In addition, a fan was installed at the top of the chamber to analyze the output characteristics according to variations in heat transfer delay. When the fan is turned ON, the air inside the chamber circulates, reducing the delay time required for heat generated by the heater to reach the temperature sensor. Table 4 summarizes the types and specifications of the experimental components and Figure 7 shows the experimental setup. The control algorithm and data acquisition process were implemented using Python 3.13.5 on a Raspberry Pi running Debian GNU/Linux 13.

### 5.2. Determination of Delay Time and Parameters

The delay time of the temperature chamber was measured for both the fan ON and fan OFF conditions. The sensing delay was defined as the elapsed time between heater activation and the first sustained temperature increase exceeding 0.1 °C for more than 3 s. With the heater continuously turned ON, the temperature data measured from the sensor were recorded three times for each condition, and the delay times were determined from the measured responses. When the fan was turned ON, the average sensing delay was approximately 45 s, whereas the average sensing delay was approximately 105 s when the fan was turned OFF.

The difference in the measured delay between the fan-ON and fan-OFF conditions is physically attributed to the heat-transfer mechanism inside the chamber. When the fan is ON, forced convection enhances air circulation and mixing, allowing heat generated by the heater to reach the sensor more rapidly. Consequently, the measured temperature responds sooner and the effective delay is reduced. When the fan is OFF, heat transport is governed mainly by natural convection and conduction, resulting in slower propagation of the thermal response and a longer effective delay. Therefore, the sensing delays were set to 50 s and 110 s, respectively, and both the initial waiting time and the minimum waiting time were set equal to the corresponding sensing delay values during the experiments. The initial heater ON duration *T_on,_*_0_ was set to 200% of the sensing delay, while the initial waiting time *T_wait,_*_0_ was set to 100% of the sensing delay. The parameter α was selected within approximately 30–50% of the sensing delay. Since these parameters are related to convergence speed and overshoot characteristics, they can be adjusted according to the desired control performance. The parameters for the hysteresis control method and the proposed control method are summarized in Table 5. For the conventional fixed dwell-time controller, the minimum residence time was set according to the experimentally measured sensing delay under each operating condition. Specifically, *T_d_* = 50 s was applied when the fan was ON, whereas *T_d_* = 110 s was applied when the fan was OFF. In each case, the dwell time was selected to approximately match the corresponding measured sensing delay, thereby providing a representative conventional lockout setting. The same hysteresis thresholds used in the conventional hysteresis experiments were applied to ensure a fair comparison. In the proposed control method, different parameter values were used for the fan ON and fan OFF conditions because the sensing delays differed between the two cases.

### 5.3. Comparison of Output and Switching Characteristics Among the Hysteresis, Fixed Dwell-Time, and Proposed Methods

**Figure 8:**
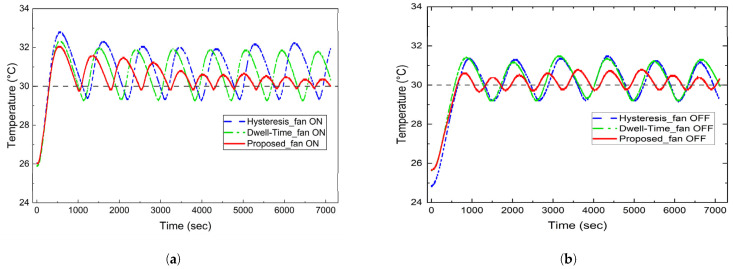
Measured temperature responses of the chamber under the hysteresis, conventional fixed dwell-time, and proposed control methods: (**a**) fan-ON condition; (**b**) fan-OFF condition.

Experiments were conducted under both fan-ON and fan-OFF conditions to compare the output responses and switching characteristics of the conventional hysteresis controller, the conventional fixed dwell-time controller, and the proposed delay-aware switching framework. The measured temperature responses are presented in Figure 8, and the corresponding performance indices are summarized in Table 6. Each condition was evaluated in a single long-duration experiment. Therefore, the reported metrics represent deterministic run-level comparisons rather than statistical averages.

As shown in Figure 8, all three control methods maintained the chamber temperature near the target value of 30 °C. However, clear differences were observed in the steady-state oscillation and switching characteristics. Under both fan conditions, the proposed framework produced smaller temperature oscillations than the hysteresis and fixed dwell-time controllers. For the fan-ON condition, the oscillation amplitudes were 2.94 °C, 2.63 °C, and 0.63 °C for the hysteresis, fixed dwell-time, and proposed methods, respectively. Under the fan-OFF condition, the corresponding values were 2.06 °C, 2.13 °C, and 0.75 °C. These results indicate that the proposed framework substantially reduced the steady-state output variation under both heat-transfer conditions.

The total number of switching transitions was calculated from the measured data collected from the start of the experiment up to 7000 s. Cases in which an additional switching event occurred within less than 10 s after a previous switching event were counted as repeated triggering events, because the thermal characteristics of the temperature chamber do not produce meaningful temperature variations within 10 s, which is significantly shorter than the sensing delay following a switching operation. The fixed dwell-time controller effectively limited switching activity by enforcing a minimum residence time after each actuator-state transition. Consequently, it produced 15 and 11 switching transitions under the fan-ON and fan-OFF conditions, respectively, which were comparable to the 14 and 11 transitions produced by the hysteresis controller. The proposed framework generated a larger number of transitions, with 25 transitions under the fan-ON condition and 20 transitions under the fan-OFF condition. Nevertheless, no repeated triggering occurred for any of the three methods in these tests. Therefore, the increased switching count of the proposed method represents regular control actions required for finer temperature regulation rather than redundant rapid switching.

The quantitative error indices further demonstrate the regulation performance of the proposed framework. Under the fan-ON condition, the steady-state root mean square error (RMSE) evaluated after 3500 s was reduced from 1.27 °C for the hysteresis method and 1.12 °C for the fixed dwell-time method to 0.37 °C for the proposed method. Under the fan-OFF condition, the corresponding RMSE values were 0.78 °C, 0.78 °C, and 0.41 °C, respectively. The proposed framework also yielded the lowest steady-state integral of absolute error (IAE) under both conditions. These results show that the conventional fixed dwell-time controller can suppress unnecessary switching through a fixed lockout interval, but this benefit is accompanied by larger temperature fluctuations and tracking errors. In contrast, the proposed framework provides a more favorable balance between switching robustness and output-regulation performance by separately regulating the actuation, protected waiting, and idle phases.

The fan-ON and fan-OFF conditions changed not only the effective sensing delay but also the thermal gain of the chamber. When the fan was turned OFF, the heat-transfer delay increased because heat propagation relied mainly on natural convection and conduction. At the same time, weaker internal air circulation reduced the fraction of heater energy reaching the sensor and increased the relative heat loss through the chamber walls.

Thus, the longer delay tended to increase the output oscillation, whereas the reduced effective thermal gain tended to decrease it. In the present chamber, the gain-reduction effect was sufficiently strong to mask part of the delay-induced increase in oscillation.

Therefore, the smaller oscillation amplitudes observed under some fan-OFF conditions should not be interpreted as evidence that a longer delay improves control performance. In a chamber with higher thermal insulation and less heat loss, the oscillation amplitude would be expected to increase more clearly with sensing delay, consistent with the simulation results presented in Section 4.

Overall, the experimental comparison confirms that the fixed dwell-time method provides effective switching suppression but sacrifices temperature-regulation accuracy because of its fixed lockout structure. The proposed framework retains the repeated-triggering prevention capability of dwell-time control while achieving substantially smaller oscillation amplitudes and steady-state errors through delay-aware phase separation and adaptive timing adjustment.

### 5.4. Comparison of the Controllers Under Different Measurement-Noise Levels

Experiments were conducted to evaluate the effects of measurement noise on the output responses and switching characteristics of the hysteresis controller, the conventional fixed dwell-time controller, and the proposed delay-aware switching framework. Because the thermal inertia of the DS18B20 sensor limits its ability to reproduce rapidly varying measurement disturbances, zero-mean Gaussian noise with standard deviations of 0.3 °C and 0.5 °C was artificially added to the measured temperature signal. These two noise levels were used to emulate different degrees of sensor uncertainty and environmental disturbance.

**Figure 9:**
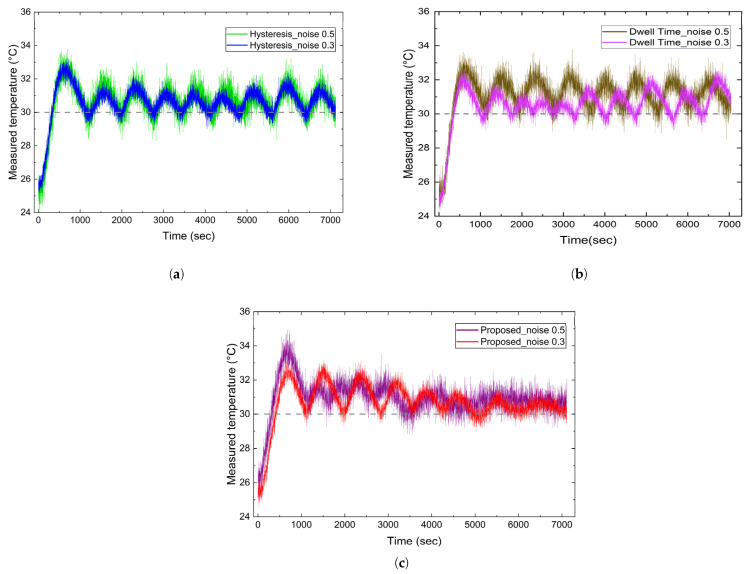
Measured temperature responses under artificially added measurement noise with standard deviations of 0.3 °C and 0.5 °C: (**a**) hysteresis control; (**b**) conventional fixed dwell-time control; and (**c**) proposed framework.

Figure 9 compares the measured temperature responses of the three control methods under the two noise conditions. In all cases, an increase in the noise standard deviation resulted in larger temperature fluctuations and higher overshoot. However, the degree to which the controllers were affected by measurement noise differed considerably. The hysteresis controller showed increasingly irregular output behavior as the noise level increased because the noisy measurement repeatedly crossed the upper and lower switching thresholds. The fixed dwell-time controller was less sensitive to rapid threshold fluctuations because actuator-state changes were prohibited during the prescribed dwell interval. Nevertheless, its fixed lockout structure resulted in relatively large and persistent output oscillations. The proposed framework also exhibited some increase in oscillation amplitude as the noise level increased, but the overall temperature response remained bounded and regular.

**Figure 10:**
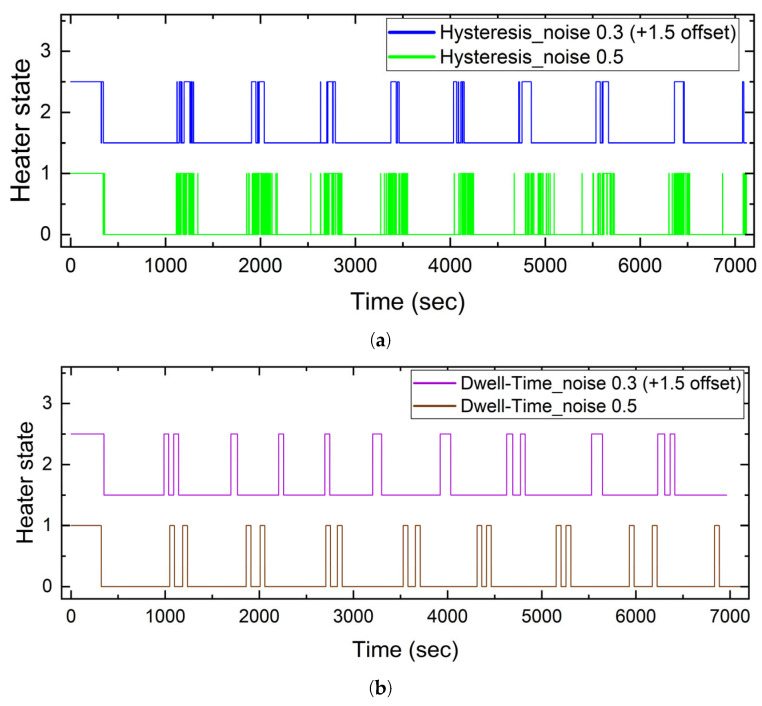

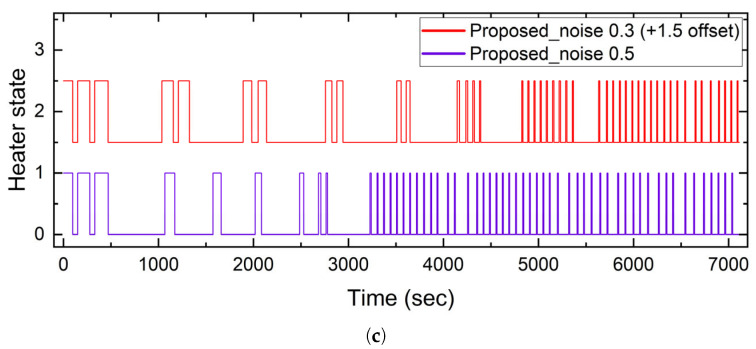
Comparison of the heater switching states obtained using the hysteresis, conventional fixed dwell-time, and proposed control methods under artificially added measurement noise: (**a**) hysteresis control; (**b**) conventional fixed dwell-time control; and (**c**) proposed framework.

Figure 10 presents the corresponding heater switching states. For the hysteresis controller, frequent clusters of rapid switching events appeared, particularly at the higher noise level. These switching events were caused by repeated threshold crossings rather than by meaningful changes in the chamber temperature. In contrast, both the fixed dwell-time controller and the proposed framework prevented rapid consecutive switching. The fixed dwell-time controller produced the smallest number of switching transitions because each actuator state was maintained for a prescribed minimum interval. The proposed framework produced more switching transitions than the fixed dwell-time controller, but the switching events remained temporally separated and no repeated triggering occurred. Therefore, the additional switching actions of the proposed framework represent regular corrective control actions rather than redundant noise-induced transitions.

The quantitative results are summarized in Table 7. For the hysteresis controller, the number of switching transitions increased from 59 at *σ* = 0.3 °C to 291 at *σ* = 0.5 °C. Among these transitions, 21 and 214 were classified as repeated triggering events, corresponding to repeated-triggering ratios of 0.36 and 0.74, respectively. This substantial increase demonstrates that the conventional hysteresis controller becomes highly sensitive to measurement noise when threshold conditions are continuously evaluated.

The conventional fixed dwell-time controller completely eliminated repeated triggering under both noise conditions. It produced only 25 switching transitions at *σ* = 0.3 °C and 31 transitions at *σ* = 0.5 °C. However, this strong switching suppression was accompanied by degraded output-regulation performance. Its oscillation amplitude increased from 3.83 °C to 4.85 °C as the noise level increased, and its steady-state RMSE increased from 1.06 °C to 1.37 °C. These results indicate that a fixed lockout interval can effectively prevent rapid switching, but may delay necessary control actions and thereby increase temperature variation.

The proposed framework also achieved zero repeated triggering under both noise conditions, while maintaining smaller output oscillations than the fixed dwell-time controller. Its oscillation amplitude increased from 2.24 °C at *σ* = 0.3 °C to 3.12 °C at *σ* = 0.5 °C, and the corresponding steady-state RMSE values were 0.70 °C and 0.92 °C, respectively. Although the proposed method generated 83 and 109 switching transitions, these values were substantially lower than those of the hysteresis controller at the higher noise level and were not associated with repeated triggering. Compared with the fixed dwell-time controller, the proposed framework therefore provided finer temperature regulation while preserving protection against rapid noise-induced switching.

The total actuator ON durations were broadly comparable among the three methods, indicating that the observed differences in output response were primarily related to switching timing rather than to a substantial difference in accumulated heater operation. The proposed framework did not minimize the number of switching transitions or the total ON duration. Instead, it achieved a more favorable balance between repeated-triggering suppression, switching regularity, and steady-state temperature regulation.

Overall, the experimental results under artificially added measurement noise confirm that conventional hysteresis control is vulnerable to repeated triggering as the noise level increases. Fixed dwell-time control effectively suppresses repeated switching, but its fixed residence-time constraint leads to larger oscillations and steady-state errors. In contrast, the proposed delay-aware framework eliminates repeated triggering while maintaining smaller oscillation amplitudes and lower steady-state RMSE through explicit phase separation and adaptive timing adjustment.

## 6. Discussion

The results show that the main advantage of the proposed framework is not the minimization of switching activity itself, but the suppression of redundant switching while preserving output-regulation performance. This distinction is important because a low switching count can also be achieved by imposing a long fixed lockout interval, which may delay necessary corrective actions and increase the output oscillation.

The hysteresis controller and the fixed dwell-time controller exhibited different limitations. The hysteresis controller became increasingly sensitive to measurement noise because continuously evaluated thresholds repeatedly responded to noisy and delayed measurements. The fixed dwell-time controller effectively prevented repeated triggering, but its fixed residence-time constraint produced larger steady-state oscillations and tracking errors. These results confirm the inherent trade-off in conventional methods between switching suppression and regulation accuracy.

The proposed framework provided a more favorable balance between these two objectives. Although it generated more transitions than the fixed dwell-time controller, the switching events remained temporally separated and represented regular corrective actions rather than noise-induced switching clusters. The smaller oscillation amplitudes and steady-state RMSE values indicate that separating the actuation, protected waiting, and idle phases allows the controller to retain dwell-time protection without relying on a single fixed lockout duration.

The total actuator ON duration was not consistently reduced in every experimental condition. Therefore, the present results should not be interpreted as demonstrating a general reduction in energy consumption. The experimentally supported benefit is the improvement in switching regularity and steady-state regulation. Potential reductions in actuator wear, switching losses, and maintenance requirements require separate long-term evaluation.

The fan-ON and fan-OFF experiments also show that the measured response was influenced by both sensing delay and thermal-gain variation. Turning the fan OFF increased the heat-transfer delay, but also reduced internal air circulation and altered the effective heat transfer to the sensor. Therefore, the experimental differences between the two fan conditions cannot be attributed to delay alone. This coupling represents a limitation of the current thermal setup. The present experiments consider fixed delay settings within each run. For a bounded time-varying delay, the framework can be conservatively implemented by selecting *T_wait,min_* ≥ *τ*_max_ + Δ_τ_. However, this may reduce responsiveness. Online delay estimation and gain-scheduled timing updates are therefore required for efficiently handling large or rapid delay variations and remain subjects of future work.

The present study assumes a fixed effective delay within each operating condition and validates the framework using a single-input thermal chamber. In addition, the adaptive analysis establishes boundedness and conditional local convergence rather than unconditional global convergence. Future work should investigate online delay estimation, time-varying delays, systematic parameter selection, repeated-trial statistical validation, and implementation in larger-scale or multi-actuator systems.

## 7. Conclusions

This paper proposed a delay-aware switching framework for binary actuator systems operating under sensing delay and measurement noise. By separating each control cycle into actuation, protected waiting, and idle phases, the framework prevents delayed or noisy measurements from immediately producing redundant switching commands. A low-complexity supervisory mechanism adjusts the timing parameters using measured tracking and overshoot information without requiring plant-model identification.

Simulation and experimental results confirmed that the proposed framework eliminated repeated triggering under the tested conditions and maintained smaller steady-state oscillations and RMSE values than the conventional fixed dwell-time controller. The hysteresis controller remained vulnerable to noise-induced repeated switching, whereas the fixed dwell-time controller suppressed rapid switching at the cost of degraded regulation accuracy.

The results demonstrate that regulating the timing of condition evaluation provides a practical compromise between switching robustness and output performance in binary actuator systems. Future studies will address time-varying delays, systematic tuning, repeated experimental validation, and extension to larger and multi-actuator applications.

## Figures and Tables

**Table 1:** Comparison of conventional switching strategies and the proposed framework.

Method	Repeated Triggering Prevention	Delay Handling	Timing Adaptation
Hysteresis	Limited under noise	No explicit handling	No
Fixed dwell-time	Effective	Indirect through fixed lockout	No
Proposed framework	Effective	Explicit protected waiting	Yes

**Table 2:** Control parameters for each method.

Hysteresis	Proposed Method
Lower band temp. = 34 °C Upper band temp. = 36 °C	*T_on,_*_0_ = 0.5 * τ, *T_wait,_*_0_ = 1.0 * τ *T_on,min_* = 0.2 * τ, *T_on,max_* = 1.5 * τ *T_wait,min_* = 1.0 * τ, *T_wait,max_* = 1.1 * τ *α* = 2 s/°C, *β*_1_ = 1 s/°C, *β*_2_ = 2 s/°C ${OS}_{ref}$ = 2.5 °C, *λ* = 0.2

**Table 3:** Performance comparison under delay/noise conditions.

Method	Switching Transitions	Repeated Triggering	Repeated Triggering Ratio	Oscillation Amplitude	Overshoot	Total ON Time
Hysteresis	33	15	0.45	3.84 °C	6.50 °C	230.3 s
Proposed	47	0	0	1.90 °C	5.53 °C	164.4 s

**Table 4:** Experimental components and specifications.

Component	Model	Manufacturer	Specification
Single-board computer	Raspberry Pi 4B	Raspberry Pi Ltd., Cambridge, UK	4 GB RAM
Temperature sensor	DS18B20	TZT teng, Shenzhen, China	
Chamber	Custom-built		350 × 150 × 210(H) mm
Heater	54B2-Z	PTCYIDU, Shanghai, China	12 V, 50 W
Cooling fan	B07NRB5J85	Usongshine, Shenzhen, China	DC 24 V
Power supply	VP600P	Antec Inc., Beijing, China	600 W
Shielding plate	PVC foam board	TPS, Hwaseong-si, Republic of Korea	140 × 68 × 5(T) mm

**Table 5:** Control parameters for each method.

Hysteresis	Fixed Dwell-Time	Proposed Framework	
Target temp. = 30.0 °C Lower band temp. = 29.5 °C Upper band temp. = 30.5 °C	Target temp. = 30.0 °C Lower band temp. = 29.5 °C Upper band temp. = 30.5 °C Fixed dwell-time (*T_d_*) Fan ON: *T_d_* = 50 s Fan OFF: *T_d_* = 110 s	Fan = ON Target temp. = 30 °C τ = 50 s *T_on,_*_0_ = 100 s *T_on,min_* = 10 s *T_on,max_* = 150 s *T_wait,_*_0_ = 50 s *T_wait,min_* = 50 s *T_wait,max_* = 150 s *α* = 25 s/°C ${OS}_{ref}\;$ = 2.5 °C *β*_1_ = 2 s/°C, *β*_2_ = 2 s/°C *λ* = 0.6	Fan = OFF Target temp. = 30 °C τ = 110 s *T_on,_*_0_ = 220 s *T_on,min_* = 50 s *T_on,max_* = 250 s *T_wait,_*_0_ = 110 s *T_wait,min_* = 110 s *T_wait,max_* = 250 s *α* = 40 s/°C ${OS}_{ref}\;$ = 2.5 °C *β*_1_ = 2 s/°C, *β*_2_ = 2 s/°C *λ* = 0.8

**Table 6:** Experimental performance comparison.

Metric	Hysteresis Fan ON	Hysteresis Fan OFF	Fixed DT Fan ON	Fixed DT Fan OFF	Proposed Fan ON	Proposed Fan OFF
Switching transitions	14	11	15	11	25	20
Repeated triggering	0	0	0	0	0	0
Repeated triggering ratio	0	0	0	0	0	0
Oscillation amplitude	2.94 °C	2.06 °C	2.63 °C	2.13 °C	0.63 °C	0.75 °C
Overshoot	2.81 °C	1.38 °C	2.31 °C	1.44 °C	2.06 °C	0.63 °C
Total ON time	1569.92 s	2880.80 s	1829.01 s	2669.82 s	1026.17 s	2424.00 s
IAE	8430.25 °C·s	6366.90 °C·s	7642.06 °C	6131.21 °C·s	4548.51 °C·s	3562.23 °C·s
IAE (after 3500 s)	3726.04 °C·s	2344.30 °C·s	3317.02 °C·s	2389.16 °C·s	1066.32 °C·s	1190.22 °C·s
RMSE	1.46 °C	1.28 °C	1.32 °C	1.15 °C	0.94 °C	0.93 °C
RMSE (after 3500 s)	1.27 °C	0.78 °C	1.12 °C	0.78 °C	0.37 °C	0.41 °C

**Table 7:** Experimental performance comparison.

Metric	Hysteresis *σ* = 0.3 °C	Hysteresis *σ* = 0.5 °C	Fixed DT *σ* = 0.3 °C	Fixed DT *σ* = 0.5 °C	Proposed *σ* = 0.3 °C	Proposed *σ* = 0.5 °C
Switching transitions	59	291	25	31	83	109
Repeated triggering	21	214	0	0	0	0
Repeated triggering ratio	0.36	0.74	0	0	0	0
Oscillation amplitude	3.42 °C	4.34 °C	3.83 °C	4.85 °C	2.24 °C	3.12 °C
Overshoot	3.20 °C	3.79 °C	2.58 °C	3.62 °C	3.17 °C	4.92 °C
Total ON time	1077.78 s	1197.60 s	1201.85 s	1092.73 s	1385.01 s	1190.71 s
IAE	6378.17 °C·s	7276.13 °C·s	6205.84 °C·s	9881.60 °C·s	7337.43 °C·s	8474.31 °C·s
IAE (after 3500 s)	2507.40 °C·s	2939.48 °C·s	3094.95 °C·s	4173.71 °C·s	2054.38 °C·s	2767.70 °C·s
RMSE	1.20 °C	1.35 °C	1.21 °C	1.65 °C	1.37 °C	1.48 °C
RMSE (after 3500 s)	0.86 °C	1.02 °C	1.06 °C	1.37 °C	0.70 °C	0.92 °C

## Data Availability

Data and code used in this study are available on request.
